# Supplementary material for: Deciphering the Role of Wnt and Rho Signaling Pathway in iPSC-Derived ARVC Cardiomyocytes by In Silico Mathematical Modeling
Source: Int J Mol Sci. 2021 Feb 18;22(4):2004. doi: 10.3390/ijms22042004 (PMC7923182; doi:10.3390/ijms22042004)
Supplement: Supplementary file 1 [file ijms-22-02004-s001.pdf]

SUPPLEMENTARY MATERIALS

Deciphering the role of Wnt and Rho signaling pathway in iPSC-derived ARVC cardiomyocytes by *in silico* mathematical modeling

Elvira Immacolata Parrotta[1†], Anna Procopio [2†], Stefania Scalise [2†], Claudia Esposito [2], Giovanni Nicoletta [2], Gianluca Santamaria [2, 3], Maria Teresa De Angelis [2,3], Tatjana Dorn [3], Alessandra Moretti [3,5], Karl-Ludwig Laugwitz [3,5], Francesco Montefusco[4], Carlo Cosentino [*, 2], and Giovanni Cuda [*, 2].

Content

Wnt Canonical Pathway Model (WCP)-(Table S1, S2, S3, System a)

Rho-kinase pathway model (RKP)-(Tables S4, S5, S6, S7, System b)

Integration of Wnt/ β-catenin and the RhoA-ROCK pathways-(Tables S8, S9, S10, System c)

List of primers

References

Supplementary Data

In this section we provide an in-depth description of the ODE-based mathematical model and of all its species, as well as the differential reactions in which each species is involved.

Model of Wnt Canonical Pathway (WCP)

In **Table S1** are reported all the species introduced in the extended Lee's model [1] . In particular, **Table S1** provides information about the full name, abbreviation used in the ODE system, and the initial conditions set in the model. **Table S2** shown all the reaction of the extended WCP model.

**Species**	**Compartment**	**Type**	**Concentration [nmol/l]**
Dshi	cytoplasm (c)	reactions	100
Dsha	cytoplasm (c)	reactions	0
APC_Axin_GSK3β	cytoplasm (c)	reactions	0
APC_Axin	cytoplasm (c)	reactions	0
GSK3β	cytoplasm (c)	reactions	50
APC	cytoplasm (c)	reactions	100
Axin	cytoplasm (c)	reactions	0
*f*APC_*f*Axin_ GSK3β	cytoplasm (c)	reactions	0
β-catenin{c}	cytoplasm (c)	reactions	0
β-catenin_*f*APC_ *f*Axin_ GSK3β	cytoplasm (c)	reactions	0
*f*β-catenin_*f*APC_ *f*Axin_ GSK3β	cytoplasm (c)	reactions	0
β-catenin{n}	nucleus (n)	reactions	0
*f*β-catenin	cytoplasm (c)	reactions	0
TCF	nucleus (n)	reactions	0
β-catenin_TCF	nucleus (n)	reactions	0
β-catenin_APC	cytoplasm (c)	reactions	0
plakoglobin_TCF	nucleus (n)	reactions	0
*g*PPAR	nucleus (n)	reactions	0
*g*TCF	nucleus (n)	reactions	0

PPAR	nucleus (n)	reactions	0
*g*Axin	nucleus (n)	reactions	0
Wnt	cytoplasm (c)	fixed	0
plakoglobin	nucleus (n)	reactions	0÷500

Table S1. Species and conditions. Overview of the dependent variables defined in the extended WCP model. The complex between two species is indicated with "_". The prefix "g" refers to the mRNA relative to each species, transcribed from the specific gene. The prefix "f" refers to the phosphorylated species. The "type" of reaction indicates the concentration relative to a given species determined either though a set of reactions, or by a fixed value. Same species present in two different compartments is considered mathematically distinct ({c} for cytoplasmatic species and {n} for nuclear ones). Concentrations are reported as [nmol/l].

**Reaction**	**Function**
Dshi → Dsha; Wnt	Dsh Activation (canonical Wnt)
Dsha → Dshi	Dsh Inactivation
APC_Axin_ GSK3β -> APC_Axin + GSK3β; Dsha	Destruction core inactivation via Dsha
APC_Axin_ GSK3β = *f*APC_*f*Axin_ GSK3β	(De) Phosphorilation Destruction core
APC_Axin + GSK3β = APC_Axin_ GSK3β	Destruction core Formation
Axin + APC = APC_Axin	Destruction core Formation
β-catenin{c} + *f*APC_*f*Axin_ GSK3β = β-catenin_*f*APC_*f*Axin_ GSK3β	β-catenin binding destruction core
β-catenin_*f*APC_*f*Axin_ GSK3β → = *f*β-catenin_*f*APC_*f*Axin_ GSK3β	Phosphorilation β-catenin
*f*β-catenin_*f*APC_*f*Axin_ GSK3β → *f*β-catenin + *f*APC_*f*Axin_ GSK3β	Release of phosphorylated β-catenin
*f*β-catenin →	Phosphorylated β-catenin degradation
→ β-catenin{c}	Synthesis β-catenin
β-catenin{c} →	Indipendent Wnt β-catenin degradation
**→ *g*Axin; β-catenin_TCF plakoglobin_TCF**	**gAxin expression**
Axin → APC	Axin degradation
**β-catenin{c} = β-catenin{n}**	**β-catenin Transport**
β-catenin{n} + TCF = β-catenin_TCF	β-catenin binding TCF
β-catenin{c} + APC = β-catenin_APC	β-catenin binding APC
**→ *g*TCF; β-catenin_TCF plakoglobin_TCF**	**gTCF expression**

*g*TCF →	**gTCF degradation**
**Plakoglobin + TCF = plakoglobin_TCF**	**Plakoglobin binding TCF**
**→TCF; *g*TCF**	**TCF synthesis**
**TCF →**	**TCF degradation**
**→ *g*PPAR; β-catenin_TCF plakoglobin_TCF**	**gPPAR expression**
***g*PPAR →**	**gPPAR degradation**
***g*Axin →**	**gAxin degradation**
**→ Axin; *g*Axin**	**Axin synthesis**
**→PPAR; *g*PPAR**	**PPAR synthesis**
**PPAR →**	**PPAR degradation**

Table S2. **Reactions in the extended WCP model. In bold are reported the reactions add to the original Lee's model.**

Reaction	Parameter	Value
Dsh activation	k_1	0.182 [l nmol^{-1} min^{-1}]
Dsh inactivation	k_2	0.0182 [min^{-1}]
Desctruction core inactivation via Dsha	k_3	0.05 [l nmol^{-1} min^{-1}]
Destruction core formation	k_4	0.0909 [l nmol^{-1} min^{-1}]
	k_5	0.909 [min^{-1}]
Destruction core formation – APC/Axin	k_6	1 [l nmol^{-1} min^{-1}]
	k_7	50 [min^{-1}]
Destruction core (De) phosphorylation	k_8	0.267 [min^{-1}]
	k_9	0.133 [min^{-1}]
$\beta - catenin$ binding destruction core	k_{10}	1 [l nmol^{-1} min^{-1}]
	k_{11}	120 [min^{-1}]
$\beta - catenin$ phosphorylation	k_{12}	206 [min^{-1}]
$\beta - catenin$ binding APC	k_{13}	1 [l nmol^{-1} min^{-1}]
	k_{14}	1200 [min^{-1}]
Axin degradation	k_{15}	0.33 [min^{-1}]
	k_{16}	98 [nmol l^{-1}]
$\beta - catenin$ synthesis	v	0.423 [nmol min^{-1} l^{-1}]
Indipendent Wnt β-catenin degradation	k_{17}	0.000257
$\beta - catenin$ transport	k_{18}	0.182 [min^{-1}]
	k_{19}	0.055 [min^{-1}]
Phosphorylated β-catenin degradation	k_{20}	0.417 [min^{-1}]
Release of phosphorylated $\beta - catenin$	k_{21}	206 [min^{-1}]
β-catenin binding TCF	k_{22}	1 [l nmol^{-1} min^{-1}]
	k_{23}	30 [min^{-1}]
Plakoglobin binding TCF	k_{24}	1 [l nmol^{-1} min^{-1}]
	k_{25}	30 [min^{-1}]

TCF synthesis	k_{26}	1 [min^{-1}]
TCF degradation	k_{27}	0.084 [min^{-1}]
gPPAR synthesis	v_{0s}	0.00884 [nmol min^{-1} l^{-1}]
	k_{1s}	0
	k_{as}	23
	n_s	3
	k_{2s}	0.00884
	k_{bs}	23
	k_{3s}	0
	k_{4s}	1
	k_{5s}	1
	k_{6s}	0
gPPAR degradation	k_{28}	0.01 [min^{-1}]
gTCF synthesis	v_{0s}	0.0061 [nmol min^{-1} l^{-1}]
	k_{1s}	0.0361
	k_{as}	23
	n_s	3
	k_{2s}	0
	k_{bs}	23
	k_{3s}	0
	k_{4s}	1
	k_{5s}	1
	k_{6s}	0
gTCF degradation	k_{29}	0.01 [min^{-1}]
gAxin synthesis	v_{0s}	0.0061 [nmol min^{-1} l^{-1}]
	k_{1s}	0.0361
	k_{as}	23
	n_s	3
	k_{2s}	0
	k_{bs}	23
	k_{3s}	0
	k_{4s}	1
	k_{5s}	1
	k_{6s}	0
gAxin degradation	k_{30}	0.01 [min^{-1}]
PPAR synthesis	k_{31}	1 [min^{-1}]
PPAR degradation	k_{32}	0.084 [min^{-1}]

Table S3. **Overview of all the parameters and their values, for the extended WCP model.**

Below are reported all the equations for the WCP extended model. Especially, each ODE describes the variation, over time, of the species concentrations [S] reported in **Table S1**. All the parameter values are reported in **Table S3**.

$$\dot{[Dsha]}V_c = V_c(k_1[Dshi][Wnt]) - V_c(k_2[Dsha])$$

$$\dot{[Dshi]}V_c = -V_c(k_1[Dshi][Wnt]) + V_c(k_2[Dsha])$$

$$\dot{[APC\_Axin]}V_c = V_c(k_3[APC\_Axin\_GSK3\beta][Dsha]) - V_c(k_4[APC\_Axin][GSK3\beta] - k_5[APC\_Axin\_GSK3\beta]) + V_c(k_6[Axin][APC] - k_7[APC\_Axin])$$

$$\dot{[GSK3\beta]}V_c = V_c(k_3[APC\_Axin\_GSK3\beta][Dsha]) - V_c(k_4[APC_{Axin}][GSK3\beta] - k_5[APC\_Axin\_GSK3\beta])$$

$$\dot{[APC\_Axin\_GSK3\beta]}V_c = V_c(k_4[APC\_Axin][GSK3\beta] - k_5[APC\_Axin\_GSK3\beta]) - V_c(k_8[APC\_Axin\_GSK3\beta] - k_9[fAPC\_fAxin\_GSK3\beta]) + V_c(k_3[APC\_Axin\_GSK3\beta][Dsha])$$

$$\dot{[fAPC\_fAxin\_GSK3\beta]}V_c = V_c(k_8[APC\_Axin\_GSK3\beta] - k_9[fAPC\_fAxin\_GSK3\beta]) - V_c(k_{10}[\beta - catenin\{c\}][fAPC\_fAxin\_GSK3\beta] - k_{11}[\beta - catenin\_fAPC\_fAxin\_GSK3\beta]) + V_c(k_{12}[f\beta - catenin\_fAPC\_fAxin\_GSK3\beta])$$

$$\dot{[APC]}V_c = -V_c(k_{13}[\beta - catenin\{c\}][APC] - k_{14}[\beta - cateni\_APC]) - V_c(k_6[Axin][APC] - k_7[APC\_Axin])$$

$$\dot{[Axin]}V_c = -V_c\left(\frac{k_{15}[Axin][APC]}{k_{16}+[APC]}\right) + V_c(([gAxin],1)) - V_c(k_6[Axin][APC] - k_7[APC\_Axin])$$

$$\dot{[\beta - catenin\{c\}]}V_c = -V_c(k_{10}[\beta - catenin\{c\}][fAPC\_fAxin\_GSK3\beta] - k_{11}[\beta - catenin\_fAPC\_fAxin\_GSK3\beta]) + V_c v - V_c(k_{17}[\beta - catenin\{c\}]) - (k_{18}V_c[\beta - catenin\{c\}] - k_{19}V_n[\beta - catenin\{n\}]) - V_c(k_{13}[\beta - catenin\{c\}][APC] - k_{14}[\beta - catenin\_APC])$$

$$\dot{[\beta - catenin\_fAPC\_fAxin\_GSK3\beta]}V_c = V_c(k_{10}[\beta - catenin\{c\}][fAPC\_fAxin\_GSK3\beta] - k_{11}[\beta - catenin\_fAPC\_fAxin\_GSK3\beta]) - V_c(k_{12}[\beta - catenin\_fAPC\_fAxin\_GSK3\beta])$$

$$\dot{[f\beta - catenin]}V_c = V_c(k_{12}[\beta - catenin\_fAPC\_fAxin\_GSK3\beta]) - V_c(k_{20}[f\beta - catenin])$$

$$\dot{[f\beta - catenin\_fAPC\_fAxin\_GSK3\beta]}V_c = V_c(k_{11}[\beta - catenin\_fAPC\_fAxin\_GSK3\beta]) - V_c(k_{21}[f\beta - catenin\_fAPC\_fAxin\_GSK3\beta])$$

$$\dot{[\beta - catenin\_TCF]}V_n = V_n(k_{22}[\beta - catenin\{n\}][TCF]) - k_{23}[\beta - catenin\_TCF])$$

$$\dot{[\beta - catenin\_APC]}V_c = V_c(k_{13}[\beta - catenin\{c\}][APC]) - k_{14}[\beta - catenin\_APC])$$

$$\dot{[\beta - catenin\{n\}]}V_n = (k_{18}V_c[\beta - catenin\{c\}] - k_{19}V_n[\beta - catenin\{n\}]) - V_c(k_{22}[\beta - catenin\{n\}][TCF] - k_{23}[\beta - catenin\_TCF])$$

$$\dot{[TCF]}V_n = -V_n(k_{22}[\beta - catenin\{n\}][TCF] - k_{23}[\beta - catenin_{TCF}]) - V_n(k_{24}[plokoglobin][TCF] - k_{25}[plakoglobin\_TCF]) + V_n(k_{26}[gTCF]) - V_n(k_{27}[TCF])$$

$$\dot{[gPPAR]}V_n = V_c\left(\frac{vos*\left(1+\left(\frac{[plakoglobin\_TCF]}{kbs}\right)^{ns}\right)}{1+\left(\frac{[plakoglobin\_TCF]}{kbs}\right)^{ns}+\left(\frac{[\beta-catenin\_TCF]}{kas}\right)^{ns}}\right) - V_n(k_{28}[gPPAR])$$

$$\dot{[plakoglobin\_TCF]}V_n = V_n(k_{24}[plakoglobin][TCF] - k_{25}[plakoglobin\_TCF])$$

$$\dot{[plakoglobin]}V_n = -V_n(k_{24}[plakoglobin][TCF] - k_{25}[plakoglobin\_TCF])$$

$$[g\dot{T}CF]V_n = V_n\left(\frac{\left(v_{0s}+k_{1s}\left(\frac{[\beta-catenin\_TCF]}{k_{as}}\right)^{n_s}\right)}{1+\left(\frac{[plakoglobin\_TCF]}{k_{bs}}\right)^{n_s}+\left(\frac{[\beta-catenin\_TCF]}{k_{as}}\right)^{n_s}}\right) - V_n(k_{29}[gTCF])$$

$$[g\dot{A}xin]V_n = V_n\left(\frac{\left(v_{os}+k_{1s}\left(\frac{[\beta-catenin\_TCF]}{k_{as}}\right)^{n_s}\right)}{1+\left(\frac{[plakoglobin\_TCF]}{k_{bs}}\right)^{n_s}+\left(\frac{[\beta-catenin\_TCF]}{k_{bs}}\right)^{n_s}}\right) - V_n(k_{30}[gAxin])$$

$$[P\dot{P}AR]V_n = V_n(k_{31}[gPPAR]) - V_n(k_{32}[PPAR])$$

System a. ODE equations in WCP extended model.

Rho-kinase pathway model (RKP)

Since no model for Rho-kinase pathway is available, we started with the identification of the reaction scheme to be included in the model definition phase.

As already discussed, the information regarding the reaction and the respective parameters for some mechanisms, such as phosphorylation and de-phosphorylation of RhoA protein and Rho-Kinase activation, were taken from literature. For all other reactions, for which no quantitative reference was found in literature, were described through simple mass-action kinetics, while their parameters were obtained through a fitting phase, keep in the physiological ranges reported in **Table S4**.

Parameter	Description	Realistic Range	Applied Range
k	Half-maximal activation coefficient	10^{-3}-10	10^{-3}-10
H	Half-life in intracellular environment	1-10^{4} min (for mRNA or protein)	5-100 min
n	Hill-coefficient	1-50 (highest measured is 35)	1-10
α	Saturability coefficient for an enhancer	1-10	1-10
transfer rates	How much reactions occurs per unit time	10^{-3}-10	10^{-3}-10
transform rates	For cleavage, phosphorylation, etc….	10^{-3}-10	10^{-3}-10

Table S4. Physiological range of kinetic parameters [2, 3].

Species and reactions considered for the model of Rho pathway are listed in**Tables S5** and **S6**, respectively.

**Species**	**Compartment**	**Type**	**Concentration [nmol/l]**
RhoaGDP	cytoplasm (c)	reactions	120
RhoaGTP	cytoplasm (c)	reactions	0
RhoGEF	cytoplasm (c)	reactions	100
RhoGAP	cytoplasm (c)	reactions	0
ROCK	cytoplasm (c)	reactions	680
pROCK	cytoplasm (c)	reactions	0
ncDsha	cytoplasm (c)	reactions	0
ncDshi	cytoplasm (c)	reactions	100
Wnt5b	cytoplasm (c)	fixed	0
Daam1a	cytoplasm (c)	reactions	0
Daam1i	cytoplasm (c)	reactions	50
*f*Actin	cytoplasm (c)	reactions	100
*g*Actin	cytoplasm (c)	reactions	0
MKL1{c}	cytoplasm (c)	reactions	1
*g*Actin_MKL1	cytoplasm (c)	reactions	0
MKL1{n}	nucleus (n)	reactions	0
*g*PPAR	nucleus (n)	reactions	0
PPAR	nucleus (n)	reactions	0

Table S5. **Overview of the dependent variables defined in the RKP model**. The complex between two species is indicated with "_". The prefix "g" refers to the mRNA relative to each species, transcribed from the specific gene. The prefix "f" refers to the phosphorylated species. The "type" of reaction indicates the concentration relative to a given species determined either though a set of reactions, or by a fixed value. Same species present in two different compartments is considered mathematically distinct ({c} for cytoplasmatic species and {n} for nuclear ones). Concentrations are reported as [nmol/l].

**Reaction**	**Function**
RhoaGDP → RhoaGTP; RhoGEF	(De) Phosphorilation
RhoaGTP → RhoaGDP; RhoGAP	(De) Phosphorilation
ROCK → pROCK; RhoaGTP	(De) Phosphorilation
pROCK → ROCK	Henri-Michaelis-Menten (irreversible)
ncDshi + Wnt5b → ncDsha	Mass action (irreversible)

ncDsha → ncDshi	Mass action (irreversible)
Daam1i → Daam1a; ncDsha	Rate law for *Daam1 Activation*
Daam1a → Daam1i	Mass action (irreversible)
RhoaGDP → RhoaGTP; Daam1a	(De) Phosphorilation
RhoaGTP → RhoaGDP	Mass action (irreversible)
*f*Actin → *g*Actin	Mass action (irreversible)
*g*Actin → *f*Actin; pROCK	Rate law for *Actin filaments Formation*
*g*Actin + MKL1{c} = *g*Actin	Mass action (reversible)
MKL1{c} = MKL1{n}	Transport
→ *g*PPAR; MKL1{n}	Gene regulation
*g*PPAR →	Mass action (irreversible)
→ PPAR; *g*PPAR	Translation
PPAR →	Mass action (irreversible)

Table S6. **Reactions used to model the Rho-kinase pathway**. The majority of reactions are expressed as mass action. Daam1 activation and conversion of Actin (from g-Actin to f-Actin and *vice-versa*) are two customized function. ncDsh*a* stands for noncanonical active Dishevelled. The majority of the reactions are expressed as reversible or irreversible mass action laws.

Reaction	Parameter	Value
Activation RhoA and ROCK and inactivation RhoA	k	0.06 [min^{-1}]
	km	100 [nmol l^{-1}]
RhoA-GTP inactivation	k_{ri}	0.0262 [min^{-1}]
Inactivation ROCK	k_2	0.6 [nmol min^{-1} l^{-1}]
N.C. Dsh activation	k_3	0.182 [l $nmol^{-1}$ min^{-1}]
N.C. Dsh inactivation	k_4	0.0182 [min^{-1}]
Daam1 activation	k_5	0.05 [l $nmol^{-1}$ min^{-1}]
Daam1 inactivation	k_6	0.262 [min^{-1}]
Actin filaments degradation	k_{act}	0.1 [min^{-1}]
Actin filaments formation	k_{actf}	0.1 [l $nmol^{-1}$ min^{-1}]
MKL1 binding	k_7	0.1 [l $nmol^{-1}$ min^{-1}]
	k_8	0.01 [min^{-1}]
MKL1 transport	k_9	0.182 [min^{-1}]
	k_{10}	0.055 [min^{-1}]
gPPAR synthesis	v_0	0.005 [nmol min^{-1} l^{-1}]
	k_a	5 [nmol l^{-1}]
gPPAR degradation	k_{11}	0.01 [min^{-1}]
PPAR synthesis	k_{12}	1 [min^{-1}]
PPAR degradation	k_{13}	0.084 [min^{-1}]

Table S7. Names and values of the parameters in the Rho-kinase pathway.

Below are reported all the equations used to model the Rho-kinase pathway. All the parameter values are reported in **Table S7**.

$$[Rhoa\dot{G}DP]V_c = -V_c\frac{k[RhoGEF][RhoaGDP]}{km+[RhoaGDP]} + V_c\frac{k[RhoGAP][RhoaGTP]}{km+[RhoaGTP]} -V_c\frac{k[Daam1a][RhoaGDP]}{km+[RhoaGDP]} + V_c(k_{ri}[RhoaGTP]) \quad \textbf{(b.1)}$$

$$[Rhoa\dot{G}TP]V_c = V_c\frac{k[RhoGEF][RhoaGDP]}{km+[RhoaGDP]} - V_c\frac{k[RhoGAP][RhoaGTP]}{km+[RhoaGTP]} + V_c\frac{k[Daam1a][RhoaGDP]}{km+[RhoaGDP]} -V_c(k_{ri}[RhoaGTP]) \quad \textbf{(b.2)}$$

$$[RO\dot{C}K]V_c = -V_c\frac{k[RhoaGTP][ROCK]}{km+[ROCK]} + V_c\frac{k_2[pROCK]}{km+[pROCK]} \quad \textbf{(b.3)}$$

$$[\,pRO\dot{C}K]V_c = V_c\frac{k[RhoaGTP][ROCK]}{km+[ROCK]} - V_c\frac{k_2[pROCK]}{km+[pROCK]} \quad \textbf{(b.4)}$$

$$[ncD\dot{s}ha]V_c = V_c(k_3[ncDshi][Wnt5b]) - V_c(k_4[ncDsha]) \quad \textbf{(b.5)}$$

$$[ncD\dot{s}hi]V_c = -V_c(k_3[ncDshi][Wnt5b]) + V_c(k_4[ncDsha]) \quad \textbf{(b.6)}$$

$$[Daa\dot{m}1a]V_c = V_c(k_5[Daam1i][ncDsha]) - V_c(k_6[Daam1a]) \quad \textbf{(b.7)}$$

$$[Daa\dot{m}1i]V_c = -V_c(k_5[Daam1i][ncDsha]) + V_c(k_6[Daam1a]) \quad \textbf{(b.8)}$$

$$[fAc\dot{t}in]V_c = -V_c(k_{act}[fActin]) + V_c(k_{actf}[gActin][pROCK]) \quad \textbf{(b.9)}$$

$$[gAc\dot{t}in]V_c = V_c(k_{act}[fActin]) - V_c\big(k_{actf}[gActin][pROCK]\big) - V_c(k_7[gActin][MKL1\{c\}] -k_8[gActin\_MKL1]) \quad \textbf{(b.10)}$$

$$[MK\dot{L}1\{c\}]V_c = -V_c(k_7[gActin][MKL1\{c\}] - k_8[gActin\_MKL1]) -V_c(k_9[MKL1\{c\}] + V_n\ (k_{10}[MKL1\{n\}])\ ) \quad \textbf{(b.11)}$$

$$[gAc\dot{t}in\_MKL1]V_c = V_c(k_7[gActin][MKL1\{c\}] - k_8[gActin\_MKL1]) \quad \textbf{(b.12)}$$

$$[MK\dot{L}1]V_n = V_c(k_9[MKL1\{c\}]) - V_n(k_{10}[MKL1\{n\}]) \quad \textbf{(b.13)}$$

$$[gPP\dot{A}R]V_n = V_n\frac{v_0}{1+[MKL1\{n\}/k_a]} - V_n(k_{11}[gPPAR]) \quad \textbf{(b.14)}$$

$$[PP\dot{A}R]V_n = V_n(k_{12}[gPPAR]) - V_n(k_{13}[PPAR]) \quad \textbf{(b.15)}$$

System b. ODE equations (b.1 - b.15) defined to model the Rho-kinase pathway.

Integrative model of Wnt/β-catenin and RhoA-ROCK pathways.

Finally, species and reactions added or changed in the WCP-RKP model are listed in **Tables S8** and **S9**, respectively.

Species	Compartment	Type	Concentration [nmol/l]
plakoglobin{c}	cytoplasm (c)	reactions	0
plakoglobin{n}	nucleus (n)	reactions	0
Dplakoglobin	cytoplasm (c)	reactions	100
Siah2i	cytoplasm (c)	reactions	100
Siaha	cytoplasm (c)	reactions	0
*f*β-catenin	cytoplasm (c)	reactions	0
*g*TCF	nucleus (n)	reactions	0
*g*Axin	nucleus (n)	reactions	0

Table S8. Overview of all the variables that have been added or changed in the WCP-RKP final model. The complex between two species is indicated with "_". The prefix "g" refers to the mRNA relative to each species, transcribed from the specific gene. The prefix "f" refers to the phosphorylated species. The "type" of reaction indicates the concentration relative to a given species determined either though a set of reactions, or by a fixed value. Same species present in two different compartments is considered mathematically distinct ({c} for cytoplasmatic species and {n} for nuclear ones). *Dplakoglobin* species refers to the desmosomal plakoglobin. Concentrations are reported as [nmol/l].

Reaction	Function
plakoglobin{c} → Dplakoglobin; pROCK	Activation with modifier
plakoglobin{c} → plakoglobin{n}	Transport
Siah2i → Siah2a; Wnt5b	Activation with modifier
Siah2a → Siah2i	Mass action (irreversible)
Siah2a + APC = Siah2a_APC	Mass action (reversible)
β-catenin{c} → *f*β-catenin; Siah2a_APC	(De) Phosphorilation
Dplakoglobin → plakoglobin{c}	Mass action (irreversible)

Table S9. Reactions used to model the WCP-RKP cross-talk.

Reaction	Parameter	Value
Activation/inactivation Dsh (c. Wnt)	k_1	0.182 [l nmol^{-1} min^{-1}]
	k_2	0.0182 [min^{-1}]
Activation/inactivation Dsh (n.c. Wnt)	k_3	0.182 [l nmol^{-1} min^{-1}]
	k_4	0.0182 [min^{-1}]
pROCK stabilizes the desmosome	k_5	0.1
Desmosomal plakoglobin	k_6	0.01 [min^{-1}]
Plakoglobin transport	k_7	0.182 [min^{-1}]
	k_8	0.055 [min^{-1}]
Plakoglobin binding TCF	k_9	1 [l nmol^{-1} min^{-1}]
	k_{10}	30 [min^{-1}]
$\beta - catenin$ binding APC	k_{11}	1 [l nmol^{-1} min^{-1}]
	k_{12}	1200 [min^{-1}]
Interaction between Siah2 and APC	k_{13}	1 [l nmol^{-1} min^{-1}]
	k_{14}	30 [min^{-1}]
Destruction core formation	k_{15}	1 [l nmol^{-1} min^{-1}]
	k_{16}	50 [min^{-1}]
Siah2 activation	k_{17}	0.1 [l nmol^{-1} min^{-1}]
Siah2 inactivation	k_{18}	0.1 [min^{-1}]

Table S10. Names and values of the parameters in the WCP-RKP cross-talk mechanism.

The mechanism of crosstalk required the addition of new differential equations in the developed mathematical system, as well as the modification of some previously ones, reported in the following **System c**. All the parameter values are reported in **Table S10**.

$$\dot{[Dshi]}V_c = -V_c(k_1[Dshi][Wnt]) + V_c(k_2[cDsha]) - V_c(k_3[Dshi][Wnt5b]) + V_c(k_4[ncDsha]) \quad \textbf{(c.1)}$$

$$\dot{[Dplakoglobin]}V_c = V_c(k_5[plakoglobin\{c\}][pROCK]) - V_c(k_6[Dplakoglobin]) \quad \textbf{(c.2)}$$

$$\dot{[plakoglobin\{c\}]}V_c = -V_c\big(k_5\big[plakoglobin\{c\}[pROCK]\big]\big) - V_c(k_7[plakoglobin\{c\}]) + V_n(k_8[plakoglobin\{n\}]) + V_c(k_6[Dplakoglobin]) \quad \textbf{(c.3)}$$

$$\dot{[plakoglobin\{n\}]}V_n = -V_n(k_9[plakoglobin\{n\}][TCF]) + V_n(k_{10}[plakoglobin\_TCF]) + V_c(k_7[plakoglobin\{c\}]) - V_n(k_8[plakoglobin\{n\}]) \quad \textbf{(c.4)}$$

$$\dot{[APC]}V_c = -V_c(k_{11}[\beta - catenin\{c\}][APC]) + V_c(k_{12}[\beta - catenin\_APC]) - V_c(k_{13}[Siah2a][APC]) + V_c(k_{14}[Siah2a\_APC]) - V_c(k_{15}[Axin][APC]) + V_c(k_{16}[APC\_Axin]) \quad \textbf{(c.5)}$$

$$[\dot{Siah2a}]V_c = V_c(k_{17}[Siah2i][Wnt5b]) - V_c(k_{18}[Siah2a]) - V_c(k_{13}[Siah2a][APC]) + V_c(k_{14}[Siah2a\_APC]) \quad \textbf{(c.6)}$$

$$[\dot{Siah2i}]V_c = -V_c(k_{17}[Siah2a][Wnt5b]) + V_c(k_{18}[Siah2a]) \quad \textbf{(c.7)}$$

$$[\dot{Siah2a\_APC}]V_c = V_c(k_{13}[Siah2a][APC]) - V_c(k_{14}[Siah2a\_APC]) \quad \textbf{(c.8)}$$

System c. ODE equations (c.1 - c.8) added to model the mechanism of crosstalk between the two pathways.

Table S11. Primers used for qRT-PCR

Gene	Primer forward	Primer reverse
GAPDH	TCCTCTGACTTCAACAGCGA	GGGTCTTACTCCTTGGAGGC
PPARγ	TGGCAATTGAATGTCGTGTC	GGAAGAAACCCTTGCATCCT
CEBPα	ACTTGGGGCTTGGAACCTAA	GACCCACGACCTAGCTTTCT

References

[1] **Heinrich R, Kirschner MW, Lee E, et al.** The Roles of APC and Axin Derived from Experimental and Theoretical Analysis of the Wnt Pathway 2003; 1; 116–32.

[2] **von Dassow G, Meir E, Munro EM OG**. The segment polarity network is a robust developmental module. *Nature* 2000; 188–92.

[3] **Read BA, Kegel J, Klute MJ, Kuo A, Lefebvre SC, Maumus F, Mayer C, Miller J, Monier A, Salamov A, Young J, Aguilar M, Claverie JM, Frickenhaus S, Gonzalez K, Herman EK, Lin YC, Napier J, Ogata H, Sarno AF, Shmutz J, Schroeder D, de Vargas C, Verret F, von GI**. Pan genome of the phytoplankton Emiliania underpins its global distribution. *Nature* 2013; DOI: doi: 10.1038/nature12221.
